# Bioconversion of L-Tyrosine into *p*-Coumaric Acid by Tyrosine Ammonia-Lyase Heterologue of *Rhodobacter sphaeroides* Produced in *Pseudomonas putida* KT2440

**DOI:** 10.3390/cimb46090603

**Published:** 2024-09-12

**Authors:** Carlos G. Calderón, Juan C. Gentina, Oscar Evrard, Leda Guzmán

**Affiliations:** 1 Molecular Biotechnology Laboratory, Biotecnos S.A., Viña del Mar 2520000, Chile; ccalderon@biotecnos.cl (C.G.C.);; 2 Fermentations Laboratory, Biochemical Engineering School, Pontificia Universidad Católica de Valparaíso, Valparaíso 2340025, Chile; carlos.gentina@pucv.cl; 3 Biological Chemistry Laboratory, Chemistry Institute, Pontificia Universidad Católica de Valparaíso, Valparaíso 2340025, Chile

**Keywords:** green chemistry, bioreactor design, fermentation optimization, bioprocess development, tyrosine ammonia-lyase, pseudomonas, *p*-hydroxycinnamic acid

## Abstract

*p*-Coumaric acid (*p*-CA) is a valuable compound with applications in food additives, cosmetics, and pharmaceuticals. However, traditional production methods are often inefficient and unsustainable. This study focuses on enhancing *p*-CA production efficiency through the heterologous expression of tyrosine ammonia-lyase (TAL) from *Rhodobacter sphaeroides* in *Pseudomonas putida* KT2440. TAL catalyzes the conversion of L-tyrosine into *p*-CA and ammonia. We engineered *P. putida* KT2440 to express TAL in a fed-batch fermentation system. Our results demonstrate the following: (i) successful integration of the TAL gene into *P. putida* KT2440 and (ii) efficient bioconversion of L-tyrosine into *p*-CA (1381 mg/L) by implementing a pH shift from 7.0 to 8.5 during fed-batch fermentation. This approach highlights the viability of *P. putida* KT2440 as a host for TAL expression and the successful coupling of fermentation with the pH-shift-mediated bioconversion of L-tyrosine. Our findings underscore the potential of genetically modified *P. putida* for sustainable *p*-CA production and encourage further research to optimize bioconversion steps and fermentation conditions.

## 1. Introduction

Phenylpropanoid acids, including cinnamic acid (CA) and 4-hydroxycinnamic, also known as para-coumaric acid (*p*-CA), have attractive biological properties for various industries, including the pharmaceutical, cosmetic, and food industries [1,2]. In the food industry, *p*-CA is used as a natural preservative, as well as to enhance certain flavor profiles due to its phenolic nature, which contributes to the astringency, bitterness, and overall flavor complexity of foods [3,4]. In sunscreens or anti-aging products, *p*-CA is an important ingredient for its antioxidant, anti-inflammatory, and UV-protective properties [5]. Due to its antioxidant activity and ability to modulate oxidative stress, this compound has therapeutic applications in treating diseases like cancer, cardiovascular diseases, and neurodegenerative disorders [6]. Its use in agricultural applications, particularly as a natural herbicide, has also been explored given its ability to inhibit the growth of certain weeds without harming crops [7].

Despite its commercial potential, the large-scale production of *p*-CA primarily relies on extraction from plant species [8,9,10,11]. However, this method faces significant challenges due to the low concentration of *p*-CA in natural sources, making it economically unsustainable. Alternative methods, such as microbial fermentation with engineered bacteria and yeast, genetic modification through metabolic engineering [12], and chemical synthesis, have been developed to address these limitations, but the extraction process remains the main approach despite its inefficiencies.

**Figure 1:**
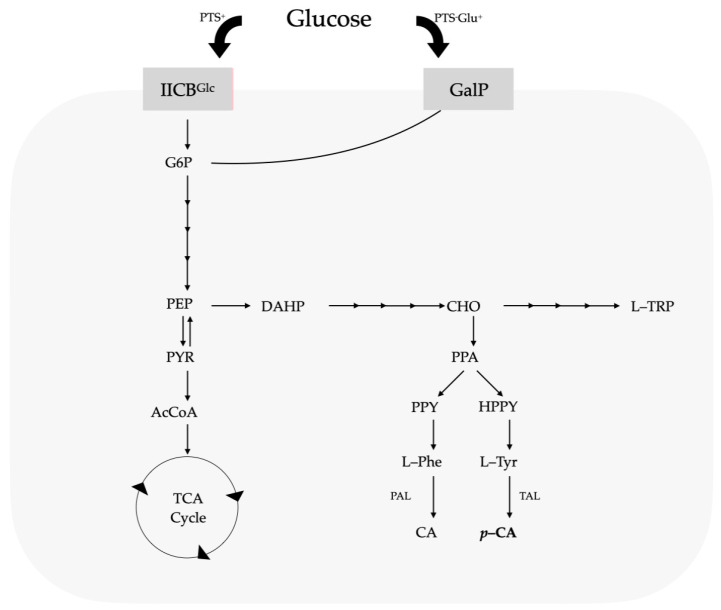
Central metabolism, carbohydrate intake routes, and aromatic biosynthetic paths. Figure adapted from Vargas-Tah, A., 2015 [27].

Ammonia-lyase enzymes are a family of proteins that catalyze the deamination of amino acids. A member of this enzyme family includes phenylalanine ammonia-lyase (PAL), which catalyzes the first stage in the phenylpropanoid route, where the deamination of L-phenylalanine (L-Phe) occurs to generate CA [11,13,14]. CA is subsequently transformed into *p*-CA via the enzyme cinnamate-4-hydroxylase (C4H) [15]. The genomes of all strains of *Pseudomonas* sequenced to date show that they have the genetic information needed to metabolize glucose [16,17,18], using three paths to convert it into 6-phosphogluconate, which is metabolized by enzymes in the Entner–Doudoroff path [19,20]. When there is any presence of succinate or other intermediaries of the tricarboxylic acids (TCAs) in the growth medium, the catabolic repression mechanisms halt bacterial glucose assimilation. The glucose intake mechanism also differs from that of other bacteria, which mainly use the phosphoenolpyruvate–carbohydrate phosphotransferase system (PTS), as seen in Figure 1.

Several of the characterized PAL enzymes can also use L-tyrosine (L-Tyr) as a substrate and thus the present activity of tyrosine ammonia-lyase (TAL) and produce *p*-CA directly from L-Tyr [21,22]. The heterologous expression of genes that encode proteins with activities (PAL/TAL) is an essential stage for generating bacterial strains to produce *p*-CA. By applying genetic engineering techniques, it is possible to develop new recombinant varieties able to synthesize phenylpropanoid acids [23,24,25]. Various microbial strains have been modified to produce CA or *p*-CA through various metabolic engineering strategies. These organisms include Gram-negative, Gram-positive, and eukaryote organisms such as *Saccharomyces cerevisiae* (*S. cerevisiae*) [26]. Producing *p*-CA through microbial processes is not straightforward due to the toxic effects of high *p*-CA concentrations in microbial cells, which can cause cell membrane damage and oxidative stress. To address these challenges, genetic engineering and the optimization of culture conditions are essential for efficient industrial-scale *p*-CA production.

For heterologous gene expression, strains *Escherichia coli* (*E. coli*), *Streptomyces lividans* (*S. lividanss*), *Pseudomonas putida S12* (*P. putida S12*), and *S. cerevisiae*, among others, have been the most extensively studied [23,24,28,29,30,31,32,33]. Sefli et al. (2021) studied *p*-CA production by generating recombinant *E. coli* using three gene sequences of TAL from *Rodobacter capsulatus* (*RcTal*), *Rodobacter sphaeroides* (*RsTal*), and *Rhodotorula toruloides* (*RtTal*), where *RtTal* was more efficient in the bioconversion of L-Tyr into *p*-CA. Integrating a copy of GroELS in *E. coli* chromosomes and optimizing culture conditions—including temperature, glycerol or lactose concentrations, and metal ion incorporation—together with the co-expressing *TrxAta*, resulted in an increased *RtTAL* activity, producing an efficient whole-cell conversion of L-Tyr into *p*-CA. *S. cerevisiae* is another microbial host that has been engineered to produce *p*-CA using extractive biphasic fermentation that is applicable in industrial fermentation processes [12]. *Corynebacterium glutamicum* (*C. glutamicum*), traditionally used in amino acid production, has also been engineered to produce *p*-CA through similar metabolic engineering approaches, such as in *E. coli* and *C. glutamicum*, and was recently engineered to produce *p*-CA from glucose. 

The aim of this study was to investigate the potential of heterologously expressing the TAL gene from *Rhodobacter sphaeroides (R. sphaeroides*) in *P. putida* KT2440 to produce *p*-CA. While *P. putida* KT2440 has not been commonly used to express TAL enzymes, the use of this strain presents several advantages to use in biotechnological process, such as HV1 certification, resilience to a wide range of organic compounds, and well-documented genetic and biochemical pathways [33,34,35,36,37,38,39,40].

A key focus of this research is optimizing the expression of specific components within the *p*-CA production pathway, following previous findings that underscore the critical role of tyrosine overproduction [41]. The selection of the TAL enzyme from *R. sphaeroides* (*R. sphaeroides*) is based on its demonstrated efficiency in converting L-Tyr into *p*-CA [42,43,44] and its inherent genetic stability, which suggests that it could sustain strong enzymatic activity under varying conditions in heterologous systems [45].

The present study also seeks to address a gap in the current literature by exploring the heterologous expression of *RsTal* in *P. putida* KT2440. The findings aim to advance our understanding of sustainable *p*-CA production through genetically engineered microbial systems integrated with bioconversion processes [24,46,47].

## 2. Materials and Methods

### 2.1. Microorganisms and Plasmids

**Figure 2:**
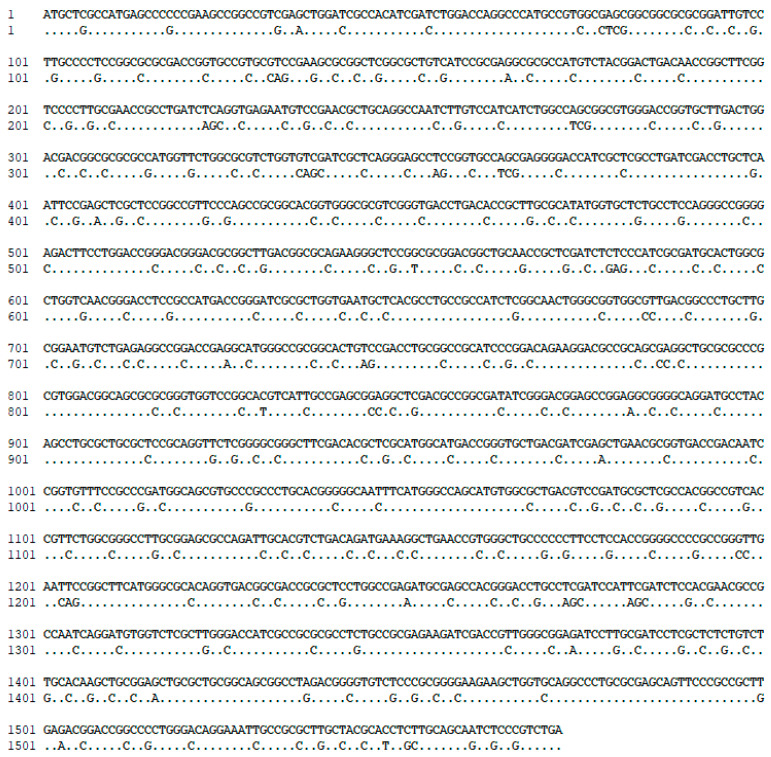
Analysis by alignment of the native *TAL* gene (full sequence) and optimized *TAL* gene (dotted sequence), with modifications expressed as nucleotide changes in given positions.

*P. putida* KT2440 (ATCC^®^ 47054^TM^) was acquired from the American Type Culture Collection (Manassas, VA, USA). The wild-type *P. putida* KT-2440 strain was grown in Luria–Bertani (LB) agar medium with Chloramphenicol, and the recombinant strain of *P. putida* KT2440 was kept in LB with Chloramphenicol and Kanamycin at 4 °C. *E. coli* TOP10 was used to maintain the plasmids generated. *RsTal* synthesis and codon optimization for *P. putida* were carried out by GenScript^®^ (Piscataway, NJ, USA) (Figure 2) in a pUC57 vector and subsequently subcloned into the pBTBX-2 vector using the restriction enzymes NcoI and XbaI (New England Biolabs, Ipswich, MA, USA). This expression vector has the characteristic of having a wide host range and is used for the expression of proteins in Gram-negative hosts (Addgene, Cambridge, MA, USA) [48]).

### 2.2. Recombinant Strain Construction

*P. putida* KT2440 was transformed by electroporation with the pBTBX-2^w^/*TAL* plasmid, then the transformant bacteria were cultivated at 30 °C in solid media prepared by the addition of 1.5% agar, and the selection of positive transformant bacteria was carried out in the presence of Kanamycin, 20 µg/mL, and Chloramphenicol, 100 µg/mL. Then, colonies were cultured in liquid culture medium in the presence of the aforementioned antibiotics and were randomly selected for plasmid extraction. The DNA plasmid extraction was performed using the QIAprep Spin Miniprep kit (QIAGEN, Hilden, Germany) according to the manufacturer’s instructions. The integrity of the purified DNA plasmid (pBTBX-2^w^/*TAL*) was run on a 1X TAE 1.0% agarose gel containing ethidium bromide and the purity of DNA obtained as assessed by the ratio of optical density 260/280 and concentration of DNA and was determined by measuring the absorbance at 260 nm using the EPOCH microplate reader (BioTek^®^ Instruments, Winooski, VT, USA). The positive transformants were verified by electrophoresis (plasmid with *TAL* gene insertion) and analyzed by enzyme restriction assay using BamHI, producing two bands around 4.1 kb (a fragment plasmid + insert) and 1.2 kb (a fragment plasmid).

### 2.3. Culture Conditions

Basic media were used [49], with the following composition: 4.70 g/L of (NH_4_)_2_SO_4_; 0.80 g/L of MgSO_4_·7H_2_O; 18.00 g/L of Na_2_HPO_4_·7H_2_O; 4.05 g/L of KH_2_PO_4_; 10 g/L of glucose; and 8mL/L of trace mix. The trace mix composition was as follows: 10 g/L of FeSO_4_·7H_2_O; 3 g/L of CaCl_2_·2H_2_O; 2.2 g/L of ZnSO_4_·7H_2_O; 0.5 g/L of MnSO_4_·4H_2_O; 0.3 g/L of H_3_BO_3_; 0.2 g/L of CoCl_2_·6H_2_O; 0.15 g/L of Na_2_MoO_4_·2H_2_O; 0.02 g/L of NiCl_2_·6H_2_O; and 1.00 g/L of CuSO_4_·5H_2_O. The antibiotics Chloramphenicol (30 g/mL) and Kanamycin (50 μg/mL) were added to the culture medium. An overnight culture was used (10 mL) to inoculate the basic medium (100 mL) in a shaker at 300 rpm and 30°C for 8 h (exponential growth phase). After incubation, bacterial cells were inoculated in a bioreactor.

The batch step was carried out in a bioreactor of 2 L capacity, using 1 L of basic medium, with glucose (40 g/L) as a carbon source and supplemented with 12 g/L of arabinose as an enzymatic production inducer. The bioreactor was inoculated with *P. putida* KT-TAL at 10% p/v of the final expected biomass in the assay, and its growth and carbon source consumption were followed until 15 h. At 15 h, 5 mL aliquots were taken from the medium to measure biomass, glucose, and arabinose. In the second stage (fed lot), feeding began to take place (after 15 h), with a glucose composition of 150 g/L. The feed flow was 17 mL/h for 18 h (306 mL). After 33 h of culture per fed lot, 2.612 grams of dissolved L-Tyr was added to an alkaline solution where the medium was adjusted to pH 8.5 with NaOH 5 N. An aliquot of protease inhibitor was added (Sigma-Aldrich, St. Louis, MO, USA). The follow-up period of the bioconversion from L-Tyr into *p*-CA was 5 h. The results for the kinetic parameters of the culture and the volumetric *p*-CA productivity were determined, and the productive period of the culture was ascertained under these conditions. For a nitrogen source and pH controller, we used a 14% (*w*/*v*) NH_4_OH solution. All experiments were performed in triplicate for each condition and repeated three times. 

### 2.4. Analytical Methods

Optical densities were measured at 600 nm (OD600) with a spectrophotometer (WPA, Cambridge, UK). CDW concentrations were calculated from OD600 values using a calibrated curve: CDW (g/L) = [OD600 × fdilution] × 0.319 + 0.077. *p*-CA concentrations were analyzed via high-performance liquid chromatography (HPLC) (1100 system, Agilent, Waldbronn, Germany) using a Zorbax 3.5 μm SB-C18 column (4.6 × 50 mm) with an acetonitrile/NaH_2_PO_4_ buffer (50 mM, pH 2, 1% acetonitrile) (25:75) as an eluent. Glucose concentrations were analyzed by HPLC (Waters Corporation, Milford, MA, USA) using an Aminex HDP-87 N column with 0.01 M Na_2_HPO_4_ as an eluent, as described before [33].

### 2.5. Statistical Analysis

Data are expressed as the mean ± SE. Statistical analysis was performed using a two-way ANOVA. A difference was considered statistically significant when *p* < 0.05. All statistical analyses were performed using GraphPad Prism 10 software (GraphPad Software, San Diego, CA, USA) 

## 3. Results and Discussion

### 3.1. Cloning and Expression

**Figure 3:**
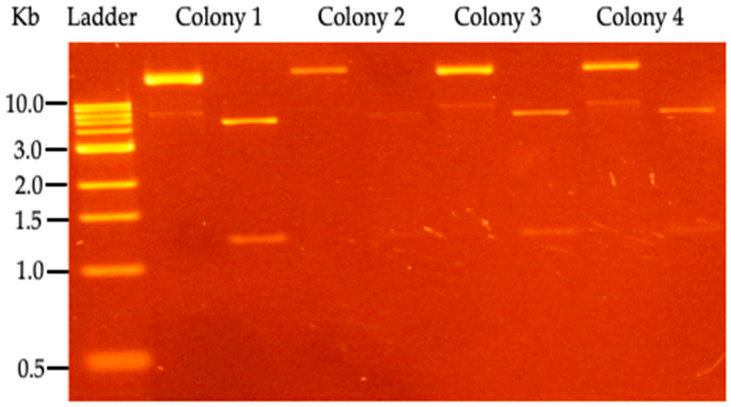
Restriction analysis of *P. putida* KT2440 clones, transformed with pBTBX-*TAL* expression vector. For each colony, the uncut vector appears in the left lane, while the right lane has the vector digested by the BamHI enzyme (NEB, Ipswich, MA, USA). 1 Kb DNA marker (NEB, Ipswich, MA, USA) was used.

Electro-transformed *P. putida* KT 2440 with a pBTBX-2^w^/*TAL* construct was tested for the ability to grow on Kanamycin; the resulting clones were digested with the BamHI enzyme. Positive colonies were selected for a TAL enzyme induction assay, and *p*-CA production was determined by HPLC analysis (Figure 3).

### 3.2. L-Tyrosine Conversion into p-CA

#### 3.2.1. *p*-CA Generation Results

Glucose/arabinose is a carbon and energy source.

**Figure 4:**
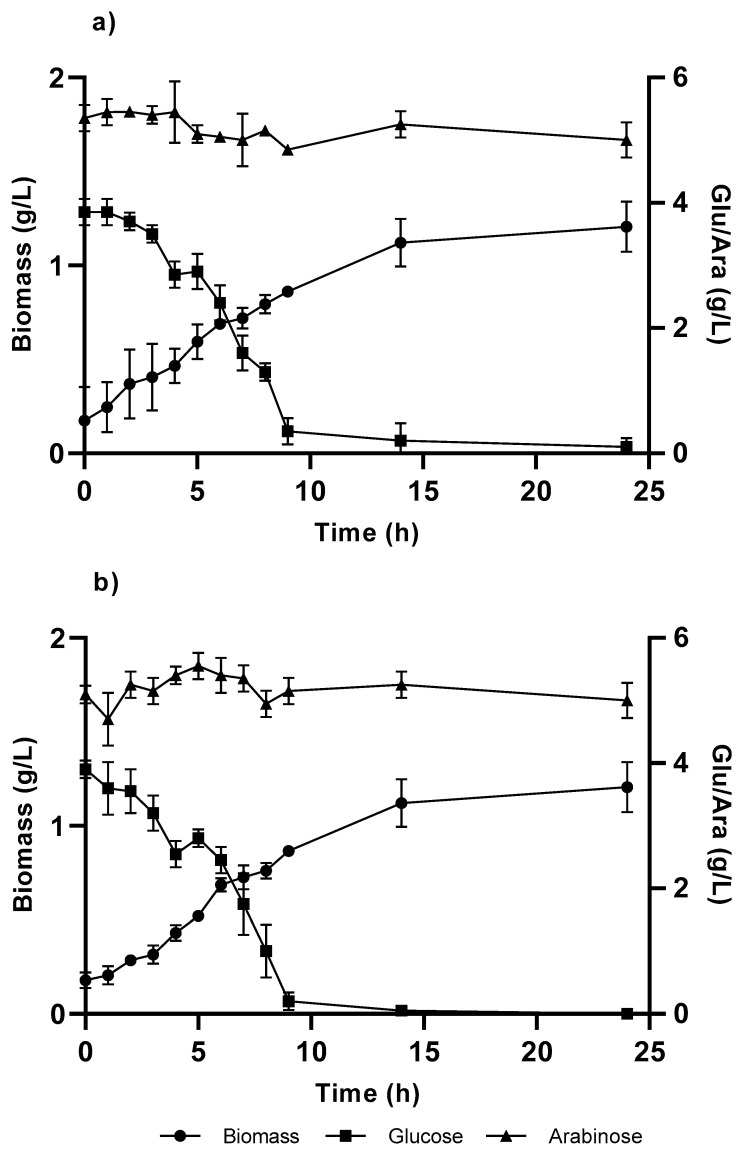
Cellular growth curve in C medium, with 4 g/L of glucose and arabinose from the *Pseudomonas putida* strains: (**a**) KT and (**b**) KT-TAL. The graph shows the results of triplicate tests.

For the growth and expression of TAL gene, it is used a combination of carbon sources consisted of glucose, which was selected for kinetic growth parameters, and arabinose, which was used as an enzymatic expression inducer for the vector pBTBX-2. Growth was performed for both strains (KT and KT-TAL). The growth profiles for biomass over time did not present major differences with each other (Figure 4), achieving a level near 1 g/L at around 14 h of culturing.

The glucose concentration decreased in the initial growth medium (4.5 g/L), causing a fall in the volumetric biomass productivities obtained in both strains compared with their growth in glucose alone, showing greater decreases at 60% in the KT strain and 58% in KT-TAL. A graphic analysis also indicates a minor alteration in the specific growth speeds (Table 1), being 20% greater for KT-TAL than when raised on glucose alone. In the other case, KT presented a µmax 5% greater than in cultures with glucose as the sole carbon and energy source.

For subsequent assays, we present the results obtained exclusively with the transformed bacterial strain since it is the one that aims to maximize its *p*-CA production.

#### 3.2.2. H_2_O/Methanol Proportion and Trifluoroacetic Acid as Extractant

Prior experiments carried out in our laboratory using a response surface methodology helped us optimize the *p*-CA extraction process using multivariable systems to find the relationship between the response and the set of independent variables used to establish the best methanol–H_2_O ratio. For this purpose, 50 mg/L standards of the antioxidant *p*-CA were used, and recovery stages were performed, where the antioxidant concentrations were quantified by HPLC, with the best extraction result obtained using 90% MeOH/10% H_2_O. Using this ratio let us obtain the highest *p*-CA production (45 mg/L), which was maintained for subsequent assays.

**Figure 5:**
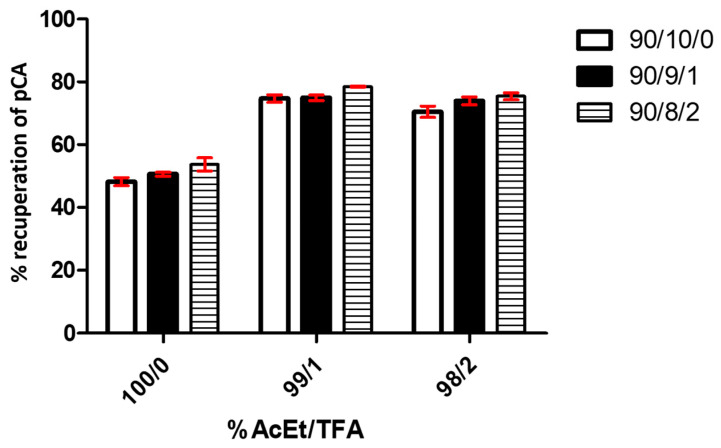
*p*-CA recuperation graphs based on different extraction processes (% AcEt/TFA) and redissolution (% MeOH/H_2_O/TFA). The graph shows the results of triplicate tests. Each bar in the graph represents different percentages of AcEt in the extraction solutions.

Figure 5 shows the results of assays that studied the effect caused by the variation in the AcEt/TFA percentage used in the extraction and the proportional MeOH/H_2_O/TFA percentage in the redissolution, where a bar graph represents the *p*-CA recuperation percentage based on a 50 mg/L pattern.

A two-way ANOVA analysis shows that the variable % of AcEt/TFA is extremely significant, affecting 95.13% of total result variance, with a *p*-value < 0.0001. The effect of TFA is clear in the recuperation performance compared with the assay without TFA (under 55% in all conditions) and with 1% of this value (above 70% in all assays). No significant difference appears between the use of 1% and 2% TFA. For its part, the variable % MeOH/H_2_O/TFA affects 2.82% of the total variance, with a *p*-value < 0.0002. It is also considered an extremely significant factor in the studied system, considering the size of the analyzed sample. The analysis determined that the interaction of both variables represented 0.26% of the total result variance, with *p* = 0.6334. We consider that the interaction between both factors is not significant in the data obtained from the experiments.

**Figure 6:**
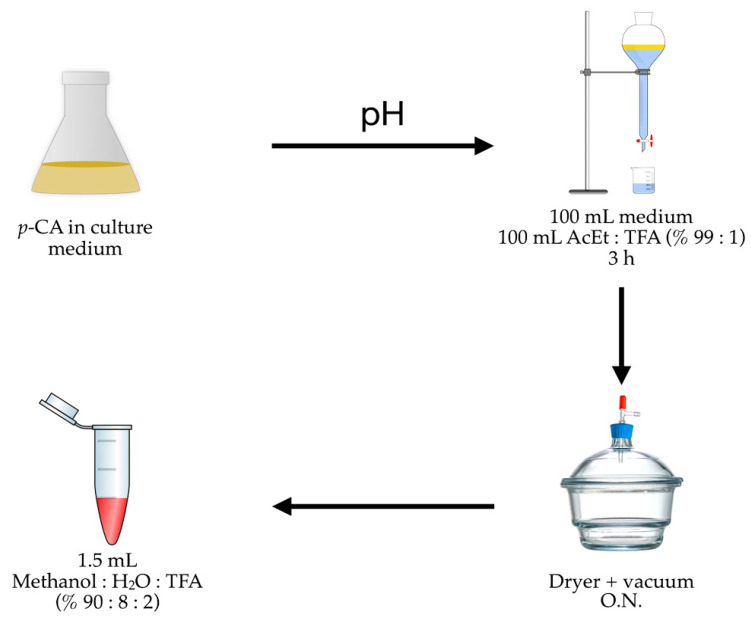
*p*-CA recuperation graphs based on different extraction processes (% AcEt/TFA) and redissolution (% MeOH/H_2_O/TFA); pH: acidification process using 2.5 M HCl.

We finally determined that the quantification of *p*-CA includes three stages (Figure 6): (i) compound extraction from the culture medium using a mixture of ethyl acetate and TFA (99:1) with agitation for 3 h; (ii) evaporating the solvent in a vacuum dryer overnight; and (iii) redissolving the *p*-CA in an aqueous methanol solution with TFA (90:8:2).

#### 3.2.3. Role of pH in L-Tyrosine Bioconversion

**Figure 7:**
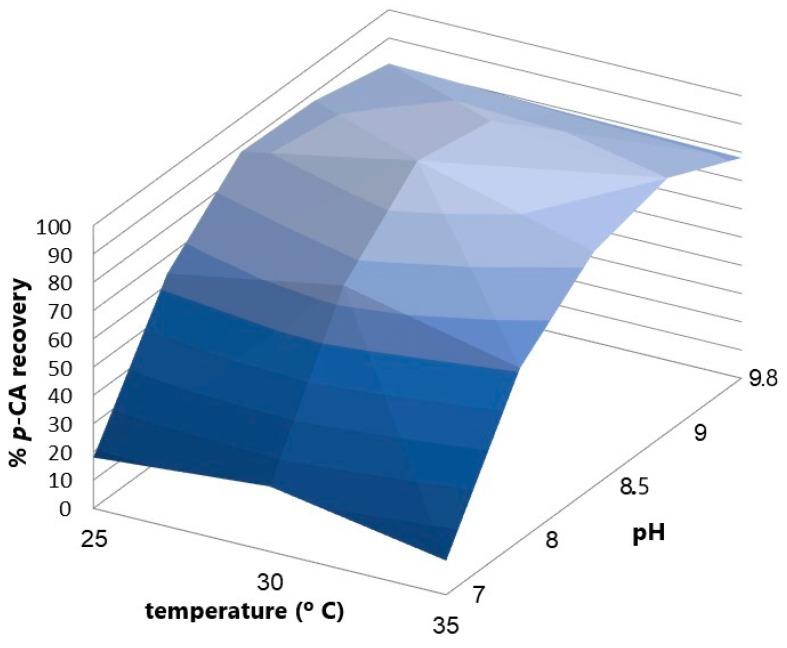
The response curve of *p*-CA recovery vs. pH and temperature variation in the enzymatic conversion process.

An experimental design with two factors, pH and temperature, was performed to find the point where both variables maximize the *p*-CA concentration produced by the bioconversion of L-Tyrosine. A battery of experiments to study the effect of pH and temperature on the conversion of L-tyrosine into *p*-CA by the KT-TAL strain (Figure 7) shows the influence of both factors on *p*-CA production using a two-way ANOVA statistical analysis. The pH factor represents around 96.65% of the total variance in the results with a *p*-value < 0.0001. Its effect is considered extremely significant. On the other hand, the temperature factor represents 1.61% of the total variance in the results (*p* < 0.0001) and is also considered extremely significant. It was determined that a pH value of 8.5 and a temperature of 30 °C maximized *p*-CA production during the bioconversion stage, which was used in later bioreactor assays.

The biomass produced in the culture by the batch in flasks was 2 g/L. These cells were used for bioconversion assays at different pH levels. The largest *p*-CA concentration obtained in the culture with pH 7 was 20 mg/L at 4 h after adding tyrosine to the medium. In turn, with the culture at pH 8.5, between 4 and 5 h, a *p*-CA level of 350 mg/L was achieved. Upon visualizing the cells of each assay with optical microscopy and a Gram stain, we clearly observed morphological changes in the bacteria. At pH 7, abundant microbial groups were noted, with a good cellular definition and size, as expected for *P. putida*. By contrast, at pH 8.5, the cells presented a decrease in size, changing to a darker color and lower density in the visual field and forming cellular agglomerations.

Based upon these assays, we conclude that pH changes helped overcome some limiting points for maximizing *p*-CA production. At pH 8.5, the catalyst was in better microenvironmental conditions than at neutral pH to perform the L-Tyr biotransformation. However, this pH made *P. putida* cells suffer a partial cellular disruption process, making it nonviable to reuse their biomass in later cycles of enzyme and *p*-CA production. With pH > 8, L-Tyr substrate solubility rises, and the bacterial cells release their cytoplasmatic content into the culture medium, leaving the active TAL enzyme available for bioconversion. 

Given the scenario, pH conditions vary between the cell growth and bioconversion stages, impeding the generation of a continual *Pseudomonas* culture process and *p*-CA production.

The pH factor represents around 96.65% of the total results variance, *p* < 0.0001, and its effect is considered extremely significant. In turn, the temperature factor represents 1.61% of the total result variance, *p* < 0.0001, which is also considered an extremely significant effect. It was determined that a pH value of 8.5 and a temperature of 30 °C maximized *p*-CA production in the bioconversion stage; these parameters were used in subsequent bioreactor assays.

Lot-mode bioreactor assays were carried out using glucose as a carbon and energy source at a concentration of 20 g/L, in 1 L of culture medium. KT-TAL strain growth was followed until 23 h of culturing. Doubling the carbon source from 10 to 20 g/L of glucose did not show any effect on the substrate performance in the biomass. Y_X/S_ remained at 0.35, which is practically equal to the biomass performance achieved in the culture by the batch in a flask (Table 2). Volumetric biomass productivity rose by 82.4% upon passing from the flask to the bioreactor. This variation is explained by external factors extrinsic to the strain, such as agitation and aeration in the reactor (via the compressor) and automatic pH and temperature controls, which allowed for better culture conditions. However, the specific maximum growth rate presented a 50% decrease compared with the flask, going from 0.21 h^−1^ to 0.11 h^−1^. This can be explained by the achievement of initial growth (first hour), which was more similar to linear relations than exponential relations (Monod-type growth) and which went on for more time, delaying the stationary phase and explaining the better performance and cellular productivity.

Culturing by batch in a bioreactor presented a clear improvement, producing the molecule of interest with regard to its maximum biotransformation behavior. This result goes together with cellular productivity since the greater the number of cells, the greater the amount of enzyme available to catalyze the conversion reaction. A larger amount of *p*-CA was formed in the bioreactors by the batch, without modifying the pH of the culture by adding L-Tyr. At 56 mg/L (non-graphed data), the result was five times greater than that obtained in flasks under the same conversion conditions. When we analyzed the production in bioreactors operated by batch, thus modifying the culture pH to 8.5 at the moment of adding L-Tyr, the difference was even greater. The maximum *p*-CA value obtained with this condition was 595 mg/L, which is 54 times the maximum value in the flask and over 10 times that obtained in a bioreactor without altering pH. These results translate into volumetric *p*-CA productivity, which was 2.3 times greater following the conversion to pH 8.5 in the reactor (Table 2). This provides experimental proof of the determining role of pH in the task of developing the biotransformation.

#### 3.2.4. Bioreactor Operated by Batch, 40 g/L of Glucose

While seeking to increase biomass concentration in the bioreactor, an assay was performed under the same conditions as previously mentioned, except for the concentration of the carbon and energy source, using 40 g/L of initial glucose. 

The development of the culture by batch was maintained for 23 h, as with the previous assay. Y_X/S_ performance was 0.33, very similar to that obtained in the reactor assay with 20 g/L of glucose (Table 2). Despite this, and as with the change from the flask to a reactor, the volumetric biomass productivity rose by 71% compared with its counterpart with a smaller amount of glucose. This represents a greater number of bacteria produced per hour, considering that the biomass generation stage was maintained for 23 h, and subsequently gave way to a biotransformation stage characterized by pH changing to 8.5 and adding L-Tyr (2 g/L). The specific maximum growth rate rose slightly to 0.13 h^−1^ compared with the assay in the bioreactor with 20 g/L of glucose. The production peak for *p*-hydroxycinnamic acid came at hour 6 after the pH change, exceeding the previous maximum production in a reactor by 22%. However, it should be noted that the volumetric productivity of the antioxidant did not maintain a linear growth relation compared with the biomass, which is explained by comparing the *p*-CA/biomass indicator. Within the bioreactor operated by batch at 20 g/L, the results were 38% higher than for this assay. The cells thus bio-transformed less L-Tyr into *p*-CA, whether due to lower TAL enzyme production or some factor that affected their activity. From the assays, we concluded that the kinetic parameters showed a favorable profile in the bioreactor run with 50 g/L of glucose. The maximum production achieved was 727 mg/L of *p*-CA at a volumetric biomass productivity of 0.53 g/L/h, slightly increasing its specific maximum growth speed to 0.13/h compared with the culture made with 20 g/L of glucose (Table 2).

#### 3.2.5. Culture by Fed-Batch and *p*-CA Production

**Figure 8:**
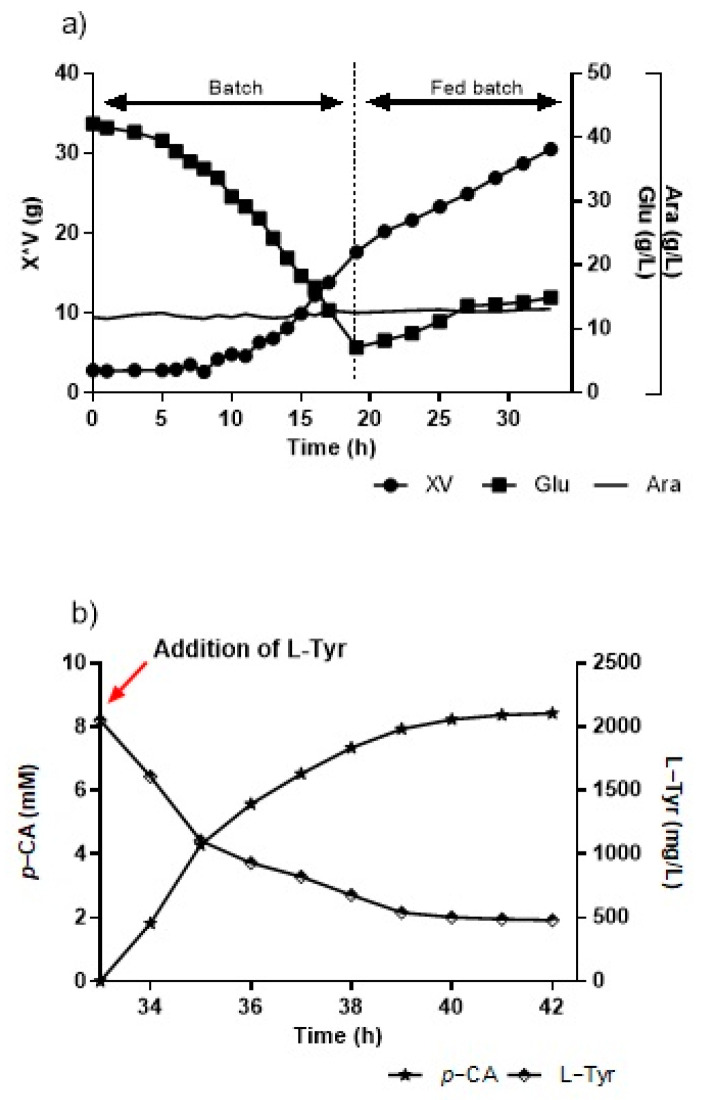
(**a**) Kinetics growth and glucose consumption in the KT-TAL strain in a bioreactor by fed-batch, with 40 g/L of initial glucose. (**b**) L-tyrosine-to-*p*-CA conversion curve in a bioreactor at pH 8.5 and 30 °C. Red arrow indicates the point of addition of L-Tyr.

For the assays carried out in a bioreactor on a fed-batch modality, we used an initial condition of 40 g/L of glucose previously defined in the batch modality. The first culture stage (batch) ran for 15 h. Upon concluding, the culture presented over 9 g/L of biomass and slightly over 15 g/L of glucose. The second stage (fed-batch) began with the feed flow, which was used at the beginning of cultivation at 15 h, with a flow of around 17 mL/h, completing the addition of around 300 mL of concentrated culture medium (300×), until 33 h are completed (Figure 8a). After 33 h, at the end of the fed-batch stage with around 25 g of cells, L-Tyr was added until a concentration of 2 g/L was reached, and pH shifted to 8.5 (Figure 8b). The production peaked at 1.16 g/L of *p*-CA at this time, while the L-Tyr substrate decreased to a little over 500 mg/L at 6 h of biotransformation. However, later analytical measurements showed that the conversion curve from L-Tyr to *p*-CA continued rising when the assay was interrupted, unlike prior assays in flasks and bioreactors where production stabilized between 5 h and 6 h.

In order to evaluate thermo-stability for the TAL enzyme within the culture medium following the pH change—where we found a mixture of proteins (including the TAL enzyme) and other cytoplasmatic residues—equivalent medium aliquots were incubated at 30 °C for between 0 and 3 h. When the enzymatic extract was incubated with a controlled temperature for 1 h, the initial maximum conversion speed of L-Tyr fell by 85% compared with the non-incubated control. At 2 h of incubation, it presented 75% of the top initial speed compared with the control, and at 3 h, it averaged 50% of the initial top speed. These results indicate that temperature influences enzyme stability and is reflected in the decreased catalytic capacity of the extract during the stages of biotransformation of L-Tyr into *p*-CA regarding to incubation time.

A second test was performed in a bioreactor with fed-batches. The difference consisted of the initial batch stage, continuing until 19 h of culturing, in order for the glucose to be consumed out of the medium down to below 10 g/L. The net biomass was around 18 g, and arabinose remained constant at around 12 g/L in all experiments. After taking the sample for 19 h, the fed-batch stage began by turning on the feeding flow from the glucose carbon and energy source at 17 mL/h via a peristaltic pump. The stage ended at 33 h with around 30 g of microbial biomass, with glucose stabilized after 26 h between 15 and 17 g/L.

## 4. Discussion

In this study, we successfully synthesized and heterologously expressed the TAL gene from *R. sphaeroides* in *P. putida* KT2440, yielding promising results in *p*-CA production. The affinity of the TAL enzyme from *R. sphaeroides* for L-Tyr compared with L-Phe, as reported in previous studies, is a key factor that facilitates the efficient bioconversion of L-Tyr into *p*-CA [50]. The specificity of the enzyme for this substrate is the key to enabling the maximization of *p*-CA production without significant interference from other L-Phe-derived products. In fact, when compared with plant-derived TAL enzymes, those from *R. sphaeroides* and *R. capsulatus* exhibit low sequence identity, with less than 32% similarity in their nucleotide and amino acid sequences [51]. This divergence may suggest an evolutionary adaptation specific to these bacteria, making them particularly interesting for industrial applications where the high-yield production of L-Tyr-derived compounds is desired. The codon optimization for TAL expression in *P. putida* is a critical step that ensures the stability and efficiency of heterologous enzyme production, contributing to achieving *p*-CA concentrations over 700 mg/L.

The specialized metabolism of *P. putida* KT2440 in aromatic compounds [52], as well as its ability to grow on hexoses like glucose [27,53], positions this strain as an ideal host for biotechnological *p*-CA production [54,55,56]. However, limitations have been observed in the use of disaccharides and pentoses [53,57], such as L-arabinose, which may influence future metabolic engineering strategies to improve process efficiency. The choice of carbon source is a determining factor in cell productivity. Our results indicate that, although glucose was not the most efficient carbon source in terms of biomass production compared with organic acids, it enabled a productivity of 0.22 g/L/h, which is consistent with previous studies [58]. However, the use of glycerol as a carbon source showed significantly lower productivity [59], suggesting that the carbon source choice must be carefully optimized depending on the production goal.

The use of the pBTBX-2 vector, which includes the pBBR replication gene for a broad host range and the pBAD/araC inducible expression system, allowed for the precise regulation of TAL gene expression in *P. putida*. However, the weak nature of the pBAD promoter, which requires high concentrations of L-arabinose to induce gene expression, represents a limitation that could be addressed in future studies by modifying the expression system to improve efficiency [48,60,61,62]. In the present study, the low efficiency of L-arabinose metabolization by Pseudomonas was a favorable characteristic given that the aforementioned pentose induces protein expression in the medium via the AraBAD operon.

It is important to note that although solvent extraction is the most commonly used method of separating cinnamic compounds, in this study, the recovery of *p*-CA was suboptimal, reaching only 50% using classical methodologies [63]. The present study was able to develop an alternative solvent extraction methodology using the most frequently described liquids from the literature to recover antioxidants such as ethanol, methanol, and ethyl acetate. However, since this is a fermentation process and not a recuperation from plant material, variations were made to maximize *p*-CA recovery from the medium. This highlights the need to develop or adapt more efficient extraction methods that are compatible with the complex matrix of a microbial culture medium. The physicochemical properties of the solvents used, along with extraction temperature and time, are critical variables that must be adjusted to maximize *p*-CA recovery and ensure the feasibility of the process on an industrial scale.

Nijkamp et al. [33] studied a *P. putida* S12 strain engineered with the PAL enzyme from *Rhodosporidium toruloides* to convert both L-Tyr and L-Phe into *p*-CA and cinnamic acid (CA), respectively. The initial flask experiments revealed that *p*-CA production was limited due to the presence of the feruloyl-CoA synthase (fcs) gene, responsible for *p*-CA degradation [52]. By interrupting this gene, Nijkamp et al. [33] achieved a significant increase in *p*-CA production, demonstrating the necessity of blocking degradation pathways to enhance yield. In this study, the focus was on biomass production and the bioconversion of L-Tyr into *p*-CA without modifying metabolic pathways, as there are no prior reports on *p*-CA production using the KT2440 wild-type strain.

Further analysis showed that pH and glucose concentration significantly influenced *p*-CA production. Flask assays with pH 7 and 8.5 indicated suboptimal conversion rates, and bioreactor experiments using different glucose levels (20 and 40 g/L) showed a 70% increase in volumetric productivity with higher glucose concentration, although the specific growth rates were lower compared with flask cultures [64]. A fed-batch strategy was employed to boost biomass and *p*-CA production with a result of 1381 mg/L, highlighting the potential of controlled feeding strategies (Table 2). However, despite achieving higher *p*-CA concentrations with the fed-batch strategy, glucose consumption efficiency was suboptimal, which limited the overall productivity. This aligns with findings from Sun et al. [49], who demonstrated that the real-time estimation of cumulative glucose consumption (CGC) could maximize bacterial biomass productivity in *P. putida* KT2440 cultures. Their method achieved a volumetric biomass productivity of 4.3 g/L/h, significantly higher than the 0.71 g/L/h obtained in the current study, suggesting that more sophisticated feeding strategies are required to optimize the fermentation process for *p*-CA production.

There is a need to explore molecular biology strategies to further enhance *p*-CA production as well. These could include engineering *P. putida* strains to overproduce L-Tyr endogenously, eliminating the need for external supplementation, and increasing the intracellular bioavailability of the amino acid. Creating strains that are resistant to the optimal pH for enzyme catalysis could also improve biomass productivity and *p*-CA yield. Other approaches—such as redirecting metabolic pathways toward L-Tyr production through the heterologous expression of phenylalanine hydroxylase (to convert L-Phe into L-Tyr) [65] or expressing C4H to convert CA into *p*-CA [66]—merit more consideration as well. These strategies could collectively lead to significant scalability improvements for *p*-CA production using *P. putida* KT2440.

## 5. Conclusions

*P. putida* KT2440 has been optimized for producing aromatic compounds like *p*-CA through metabolic engineering, which enhances its ability to tolerate and metabolize these chemicals. This study evaluated the performance of genetically modified *P. putida* strains expressing the *TAL* gene, using glucose and glycerol as carbon sources. Glucose showed higher productivity, making it the preferred carbon source for advanced fermentations.

While *P. putida* KT2440 could not degrade arabinose, this sugar effectively induced TAL gene expression via the Ara promoter, with 12 g/L arabinose being optimal for maximizing expression. A recombinant *P. putida* strain was developed to express the TAL enzyme from *R. sphaeroides*, with bioconversion initiated by adjusting the pH and adding L-tyrosine.

These findings provide a foundation for further research into optimizing *P. putida* KT2440 as a biotechnological platform for producing aromatic compounds through metabolic engineering and bioprocess optimization.

## Figures and Tables

**Table 1:** Kinetic parameters for KT and KT-TAL strains cultured in C/Glu-Ara medium.

	KT	KT-TAL
P_VX_ (g/L/h)	0.08	0.07
Y_X/Glu_	0.25	0.25
µ_max_ (h^−1^)	0.21	0.37

**Table 2:** Summary of principal kinetic parameters in the growth of recombinant KT2440 (KT-TAL) and *p*-CA production under different culture modes.

Conditions	Y_X/S_	P_VX_ (g/L/h)	μ_max_ (h^−1^)	[*p*-CA]_max_ (mg/L)	P_VP_ (mg/L/h)
Batch, 20 g/L glu	0.35	0.31	0.11	595	21
Batch, 40 g/L glu	0.33	0.53	0.13	727	25
Fed-batch, 40 g/L glu	0.28	0.71	0.17	1381	33

## Data Availability

Data sets generated for this study are available upon request from the corresponding author.
